# Presence of a mouse mammary tumour virus-like in feline lymphomas: a preliminary study

**DOI:** 10.1186/s13027-022-00449-9

**Published:** 2022-06-23

**Authors:** Francesca Parisi, Francesca Lessi, Michele Menicagli, Prospero Civita, Romano Liotti, Francesca Millanta, Giulia Freer, Mauro Pistello, Chiara Maria Mazzanti, Alessandro Poli

**Affiliations:** 1 Dipartimento di Scienze Veterinarie, Università di Pisa, Viale delle Piagge, 2, 56124 Pisa, Italy; 2 Fondazione Pisana per la Scienza, Pisa, Italy; 3 School of Pharmacy and Pharmaceutical Sciences, College of Biomedical and Life Sciences, Cardiff University, Cardiff, UK; 4 Ricerca Traslazionale e delle Nuove Tecnologie in Medicina e Chirurgia, Università di Pisa, Pisa, Italy

**Keywords:** MMTV-like virus, Feline lymphoma, Immunohistochemistry, Fragment analysis, Heminested PCR

## Abstract

The mouse mammary tumour virus (MMTV) is implicated in the aetiology of murine mammary carcinomas and a variant of it, the type B leukemogenic virus, can cause murine thymic lymphomas. Interestingly, a MMTV-like virus is suspected to be involved in human breast cancer and feline mammary carcinomas. However, to date, no cases of MMTV-like sequence amplifications have been described in lymphoid neoplasms in veterinary literature. The aim of this study was to investigate the presence of *env* nucleotide sequences and protein 14 (p14) of a MMTV-like virus in fifty-three feline lymphoma samples. Our results show that MMTV-like sequences were detected in 5/53 tumours (9.4%): three gastrointestinal lymphomas (one B-type diffuse large, one B-type small non-cleaved, and one T-type diffuse mixed lymphoma); and two nasal lymphomas (one B-type diffuse small cleaved lymphoma and one B-type diffuse mixed lymphoma). P14 expression was detected in the cytoplasm, and rarely in nuclei, exclusively of neoplastic cells from PCR-positive tumours. The correlation between the presence of the MMTV-*env* like sequences (MMTVels) and p14 antigen was statistically significant in nasal lymphomas. All cats with MMTVels-positive lymphoma had a history of contact with the outdoor environment and/or catteries, and two deceased subjects shared their environment with cats that also died of lymphoma. In conclusion, this study succeeds in demonstrating the presence of MMTVels and p14 in feline lymphomas. The characterization of the immunophenotype of MMTVels-positive lymphomas could contribute to the understanding of a possible role of a MMTV-like virus in feline tumour aetiology. The significant association between the presence of the viral sequences in lymphoid tumours and their nasal localization, together with the data collected through supplementary anamnesis, should be further analysed in order to understand the epidemiology of the virus.

## Introduction

The mouse mammary tumour virus (MMTV) is a B-type retrovirus of the Retroviridae family that causes mammary tumours in mice and has been implicated in the aetiology of murine lymphomas. It is classified as a slow-acting oncogenic retrovirus that causes mammary tumours in mice after a long latency (6 to 9 months) [1, 2]. Moreover, a MMTV variant, type-B leukemogenic virus (TBLV), has been demonstrated to cause murine thymic lymphomas with a short latency period (1.5 to 2 months) when injected intrathymically into neonatal mice [3, 4]. Infection has been deemed likely to occur in the same way as in the mammary epithelium [5]. Briefly, MMTV is shed in mothers’ milk and is transmitted from infected mothers to suckling pups through the intestinal epithelium. The virus first infects dendritic cells and B lymphocytes in Peyer’s patches of the gut [6–8]. B lymphocytes and dendritic cells process the viral superantigen (Sag) protein, express it on their cellular surface in association with class II major histocompatibility complex (MHC) and present the antigen to CD4+ T cells. Sag-activated T cells proliferate and produce cytokines, stimulating and recruiting additional dendritic, B, and T cells. In this way, the expansion of different cell clones takes place, and the result is the proliferation of both infection-competent and infected cells. In order to reach the mammary gland, the virus subverts cells of the immune system, particularly B and T cells, to establish infection [8, 9]. It is clear that both B and T lymphocytes play critical roles in MMTV infection. MMTV-infected lymphocytes carry the virus to the mammary gland [8], where mammary gland cells are infected during their proliferation phase, which mostly occurs during puberty and pregnancy [8]. The transforming activity of the virus is restricted to the mammary gland, but it is known that MMTV infection also takes place in a variety of other tissues, including the salivary gland, kidney, lung, seminal vesicle, epididymis, and testis [10–13]. High levels of MMTV expression were detected in Leydig cell tumours [14] and lymphoid leukaemia of mouse GR [15] and DBA/2 [16] strains. Whether expression of MMTV in these tumours promotes tumour development is unknown [17].

The identification of MMTV-like sequences and antigens in human mammary carcinomas has suggested that a virus homologous to MMTV may be involved in human mammary carcinoma development as well [18, 19]: case reports of women diagnosed with both lymphoma and breast cancer led to hypothesize a common aetiology for these tumours, suggesting that MMTV is involved in both breast cancer and lymphoma aetiology in humans [20]. In addition, the presence of sequences of a MMTV-like virus has been demonstrated for other animal species as well. They have been detected in rhesus macaque genomic DNA [21] and in certain feline mammary tumours [22–24], even if the role of the virus in the induction of mammary tumours in these species has not been demonstrated yet. Interestingly, it has been observed that a MMTV variant isolated from mice can be induced to productively replicate in both canine (Cf2Th) and human (HP574) cells by serial passages [25].

To date, no MMTV-like sequences have been described in lymphoid neoplasms in the veterinary literature, except for mouse lymphoma. The above-mentioned findings from human studies, together with the detection of MMTV-*env* like sequences (MMTVels) in certain types of feline mammary carcinoma and of feline inflammatory cells positive for the presence of p14, the signal peptide of the MMTV envelope protein precursor [23], led to our interest in testing feline lymphoma samples for the presence of MMTV. The present study is aimed at investigating the presence of *env* sequences and the expression of p14 in fifty-three feline lymphoma samples from different anatomic localizations.

## Materials and methods

### Animals and tissues

Fifty-three formalin-fixed paraffin-embedded (FFPE) feline lymphoma samples were selected from the Tumour Registry of the Department of Veterinary Science of the University of Pisa (2016–2020). Samples (5 from lymph nodes and 48 from other anatomical regions: 34 from gastrointestinal district, 4 from skin, 3 from nasal cavities, 2 from kidney, 2 from mediastinum, 1 from the eye, 1 from the ear canal, and 1 from trachea) were obtained after surgery or endoscopic exam from veterinary clinics in and around Pisa, Italy. Medical history and anamnesis were taken, including breed, age, and sex. An in-depth remote anamnesis was then collected for MMTVels-positive lymphomas: pet owners and referring clinicians were asked for an analysis of the sanitary conditions, the environment in which subjects had spent their lives, and anamnestic information on other pets that shared their environment with the positive ones. Normal lymphoid tissues were collected during routine necropsy with the owners’ consent from 5 cats who had died due to causes unrelated to generalized infection or lymphoid tumours.

### Molecular analysis

#### DNA extraction

Four 10-µm tissue sections from each tumour sample were kept overnight in lysis buffer containing proteinase K. DNA was extracted using Maxwell 16 FFPE DNA Purification Kit according to the manufacturer's protocol (Promega, Madison, WI). To check for cross-contamination, blank DNA samples (water) were processed in parallel with the tissue samples. The concentration of DNA was determined by Qubit Fluorometer (Life Technologies, Carlsbad, CA) with dsDNA BR assay.

#### Detection of MMTVels by fluorescence heminested polymerase chain reaction (PCR)

Fluorescent heminested PCR was used to detect the presence of MMTVels using primers based on the sequence available in GenBank (accession no. AF243039) and designed so as to generate amplicons of 201 bp or less, to ensure amplification from paraffin-embedded tissues. The outer primers yielded a 201-bp fragment from nucleotide positions 231 to 431 of MMTV-like *env*, and the inner primers yielded a 191-bp fragment (nucleotide positions 241 and 431). The sequences of the outer primers were:

- forward, 5 = -GATGGTATGAAGCAGGATGG-3 =;
- reverse, 5 = -AAGGGTAAGTAACACAGGCA-3 =.

Inner amplification was performed with the same outer reverse primer and forward primer 5 = - AGCAGGATGGGTAGAACCTA-3 =.

Both PCR amplifications were performed in a 50-μl mixture containing 5 μl of 10X Taq buffer, 4 μl of deoxyribonucleoside triphosphates (dNTPs) mixture (2.5 mM for each dNTP), 2 μl of 10 μM each unlabelled reverse primer and 6-FAM-labelled forward primer (Applied Biosystems, Milan, Italy), and 0.25 μl Takara Ex Taq DNA polymerase (Takara Biotechnology, Dalian, China). Input target template was 200 ng genomic DNA in the first round PCR and 2 μl of first-round PCR product in the second round. Thermocycler conditions for PCR were initiated with denaturing at 94 °C for 10 min, followed by 45 cycles of 95 °C for 45 s, 60 °C for 45 s, and 72 °C for 1 min, and this was ended with 72 °C for 10 min. The second run of PCR using inner primers followed the same conditions as those for the first run. The integrity and the quality of the DNA extracted were validated by PCR amplification of *gapdh* gene.

To exclude PCR contamination, water controls and negative DNA samples were included every five samples in each run. As a positive control, DNA from MMTV-positive murine mammary carcinoma cell line Mm5Mt was used. Fluorescent amplicons were analysed by capillary electrophoresis and appeared as peaks in an electropherogram. The amplicon size was extrapolated from a molecular size ladder resuspended in PCR buffer and run in parallel. Briefly, 2 μl of PCR products from amplification rounds were mixed with 0.5 μl of LIZ labeled size standard (GeneScan™ 600 LIZ, Life Technologies, Carlsbad, CA) and 9.5 μl of formamide (Hi-Di Formamide, Life Technologies, Carlsbad, CA, USA). After denaturation at 95 °C for 3 min, samples were loaded onto a 3500 Genetic Analyzer and analysed using GeneMapper software, version 3.1 (Applied Biosystems, Foster City, CA, USA).

#### Detection of murine DNA

The presence of contaminating mouse DNA was excluded by performing murine mitochondrial DNA and intracisternal A-particle (IAP) LTR PCR, according to Robinson et al. [26].

### Pathological investigations

#### Histopathology

Four-μm-thick sections were cut from feline lymphoma samples, dewaxed in xylene for 5 min, rehydrated using graded alcohols (100, 90, and 70%) and brought to water and stained with haematoxylin and eosin (HE). Lymphomas were classified according to the National Cancer Institute Working Formulation (NCIWF, 1982) [27].

#### Immunohistochemistry

Immunohistochemistry was performed to assess the immunophenotype (IP) of lymphomas and positivity for p14, the signal peptide of the MMTV envelope protein precursor. To investigate the IP, two 4-μm-thick serial sections from FFPE lymphomas were cut, dewaxed in xylene for 5 min, rehydrated using graded alcohols (100, 90, and 70%) and brought to water. Antigen retrieval was achieved by placing the slides in a bath of citric acid (pH 6.0) for sections labelled with anti-CD3 antibody and in a bath of TRIS–EDTA (pH 9.0) for those labelled with anti-CD79a antibody, and boiling them in a microwave oven for two cycles, the first one at 1000 W for 4 min and the second one at 200 W for 8 min. The slides were dried at room temperature for 20 min and washed with running tap water. Non-specific binding was prevented by incubation with 10% normal horse serum for 10 min. Afterward, samples were incubated with 1:200-diluted rabbit polyclonal anti-human CD3 antibody (Dako, Glostrup, Denmark) and 1:100-diluted mouse monoclonal anti-human CD79a (Clone HM57, Santa Cruz Biotechnology, CA, USA) for one h at room temperature. A biotin-conjugated horse anti-Mouse/Rabbit IgG antibody (H + L) R.T.U. (Vectors Laboratories, CA, USA) was applied. Antibody binding was detected using a streptavidin–biotin–peroxidase kit, Horseradish Peroxidase Streptavidin (Vector Laboratories, CA, USA). 3,3′-diaminobenzidine (0.05% for 10 min at room temperature) was used as a chromogen (Vector Laboratories, CA, USA). Stained slides were counterstained in haematoxylin for 50 s, followed by a wash in tap water, dehydrated in graded alcohols (70, 90, and 100%), and clearedwith xylene. Sections were mounted in DPX (08600E; Surgipath Europe, UK). As a positive control, a normal feline lymph node was used; as a negative control, the primary antibody was replaced with an irrelevant, isotype-matched antibody to control for secondary antibody non-specific binding. Cytoplasmic staining was considered positive. Staining intensity was not considered to assess positivity. The lymphomas were classified as either T-cell type (> 60% of cells reacting with anti-CD3 antibody) or B-cell type (> 60% of cells reacting with anti-CD79a antibody). Non-B non-T cell lymphoma were characterized by cells morphologically consistent with lymphocytes but lacking B-cell and T-cell specific antigens [28]. The assessment of positive cells was performed by three pathologists (FP, FM and AP). Six representative adjacent non-overlapping 100,000 μm^2^ fields were acquired at 400× magnification using a 10× ocular lens and 40× objective lens. Digital images were acquired using a semi-automated image analysis system (LAS 4.10, Leica, Heerbrugg, Switzerland).

To immunolocalize MMTV-like p14, an immunohistochemical staining method, previously used to stain human and feline breast cancer samples [18, 23, 24] and validated by fluorescence in situ hybridization (FISH) analysis, was used [29]. Four- μm-thick sections were dewaxed in xylene and rehydrated using graded alcohols and brought to water. Antigen retrieval was performed by microwaving sections for 9 min in citrate/EDTA buffer (pH 7.8). Non-specific peroxidase activity was blocked with 3% hydrogen peroxidase for 15 min, and non-specific binding was prevented by incubation with normal goat serum for 10 min. Afterwards, incubation with 1:2000-diluted anti MMTV-p14 rabbit polyclonal antibody (kindly provided by Dr J. Hochman, University of Jerusalem, Israel) was performed for 2 h at room temperature. Negative controls included omission of the primary antibody. A secondary biotin-conjugated goat antibody was applied, followed by enzyme-labelled streptavidin and substrate chromogen (rabbit-specific HRP/DAB-ABC detection IHC kit, ab 64261 Abcam, Italy). Slides were counterstained with haematoxylin. Cytoplasmic/nuclear staining for MMTV-p14 was considered to indicate positivity.

### Statistical analysis

The Fisher exact test analysis was used to determine if there was a correlation between the identification of MMTV and data from signalment, anatomical localization, grade of malignancy and immunophenotype of lymphomas. A *p* value less than 0.05 was significant.

## Results

### Animals

The feline breeds of the 53 cats with lymphoma included European shorthair (n = 49, 30 males, 16 females, 3 unknown), Siamese (n = 2, both males), one Norwegian Forest male, and one Carthusian male. Median age of the examined cats was 9.5 ± 3.6 years, ranging from 7 months to 14 years. Median age of the six controls was 6.3 ± 2.3 years.

### Pathological investigations and immunophenotyping

**Fig. 1:**
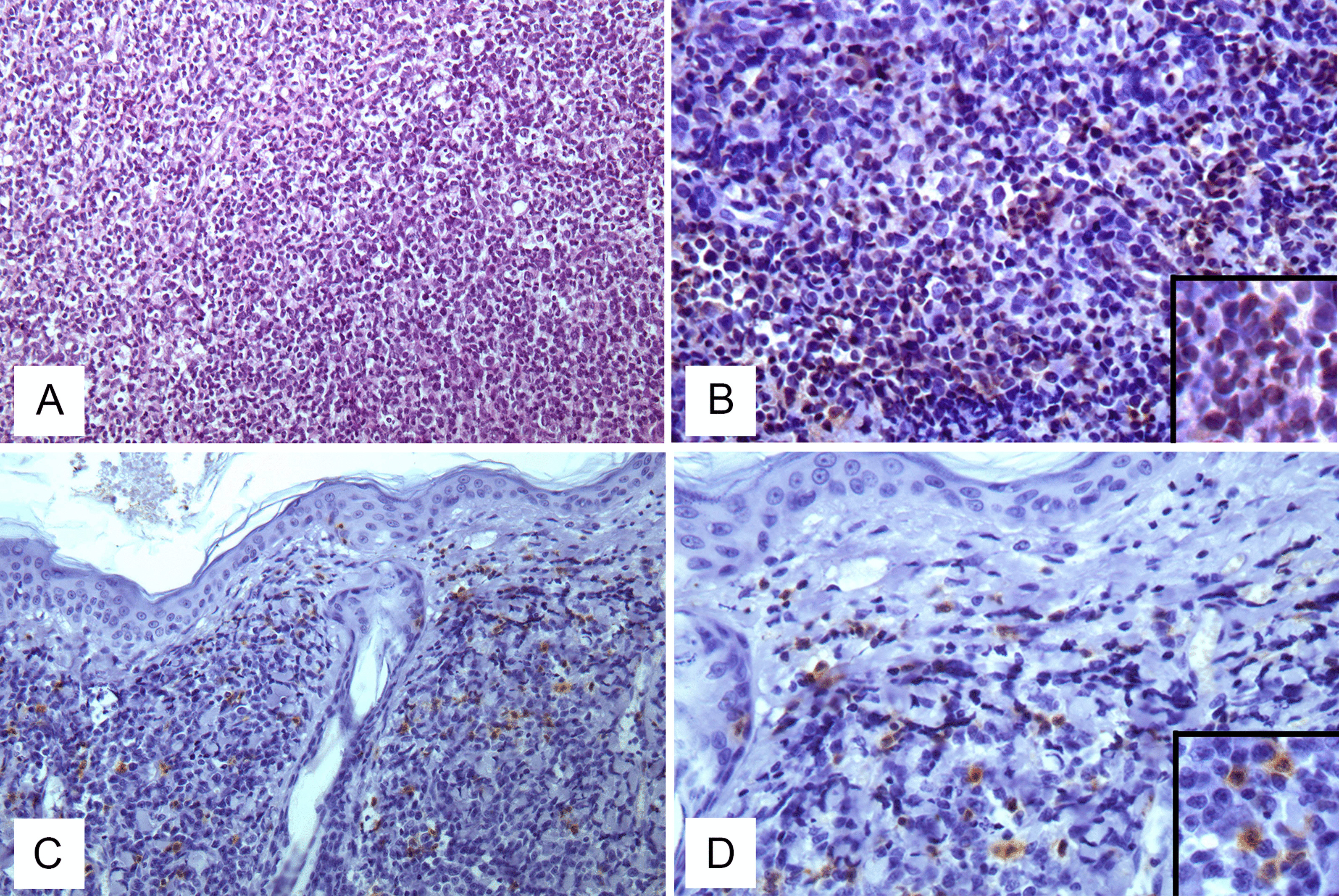
Patterns of the nodal and cutaneous feline lymphomas collected. **A**, **B** T-cell nodal lymphoma: **A** Nodal architecture of lymph node effaced by an expanding, diffuse proliferation of intermediate-sized lymphocytes (H-E stain, Ob. 20×); **B** Neoplastic lymphoid cells positively stained by anti-CD3 antibody (IHC, Ob. 40×). Insert: Cytoplasmatic staining of CD3-positive lymphocites (IHC). **C**, **D** Cutaneous T-cell lymphoma: **C** Dense, monomorphic, diffuse infiltration of CD3+  lymphocytes in the derma of the skin. Lymphocytes are found within the epithelium (IHC, Ob. 20×); **D** Strong immunostaining with anti-CD3 antibody (IHC, Ob. 40×). Insert: Cytoplasmatic staining of CD3-positive lymphocytes (IHC)

Collected lymphomas were classified as 34 gastrointestinal lymphomas (17 B-type, 13 T-type, 4 non-B and non-T type); 5 nodal lymphomas (3 B-type and 2 T-type) (Fig. 1A, B); 4 cutaneous lymphomas (1 B-type and 3 T-type) (Fig. 1C, D).

**Fig. 2:**
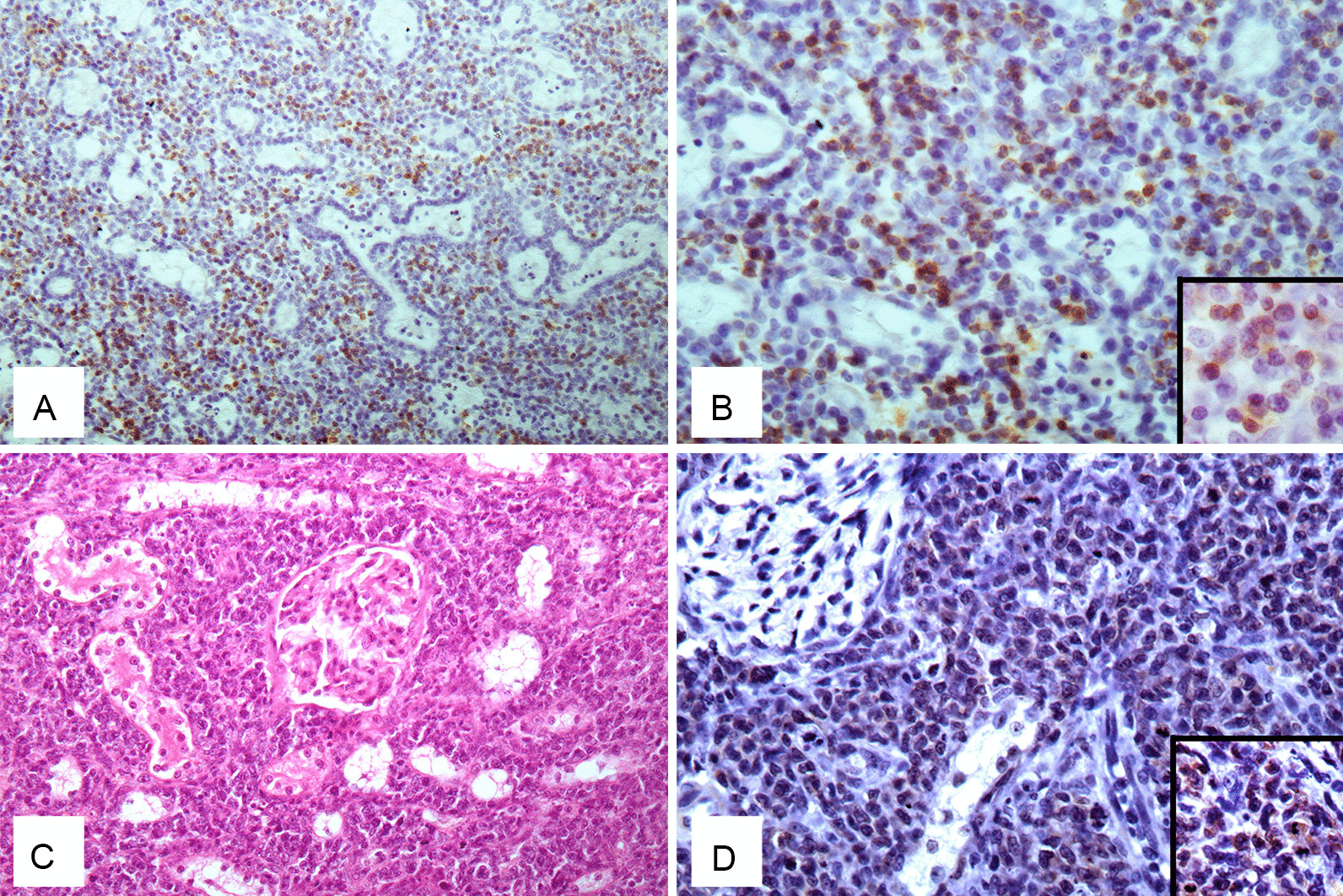
Patterns of the nasal and renal feline lymphomas collected. **A**, **B** Nasal B-cell lymphoma: **A** Dense, monomorphic, diffuse infiltration of CD79a+ lymphoblasts in the submucosal layer of the nasal cavity (IHC, Ob. 20×); **B** Diffuse, strong immunostaining with anti-CD79a antibody (IHC, Ob. 40×). Insert: Cytoplasmatic staining of CD79a-positive lymphocites (IHC). **C**, **D** Renal B-cell lymphoma: **C** Severe, monomorphic, diffuse infiltration of lymphoblasts in renal interstitium (H-E stain, 0b. 20×); **D** Diffuse, mild immunostaining of lymphoblast with anti-CD79a antibody (IHC, 0b. 40×). Insert: Cytoplasmatic staining of CD79a-positive lymphocites (IHC)

Three tumours were nasal B-type lymphomas (Fig. 2A, B); 2 renal B-type lymphomas (Fig. 2C, D); 2 mediastinal T-type lymphomas; 1 ocular B-type; 1 auricular T-type lymphoma; and 1 tracheal non-B non-T lymphoma.

According to the NCIWF classification scheme, collected lymphomas were classified as 2 (4%) low-grade lymphomas, both diffuse small lymphocytic (DSL) lymphomas, 45 (85%) intermediate-grade lymphomas, 16 diffuse small cleaved (DSC) lymphomas, 11 diffuse mixed (DM) lymphomas, 18 diffuse large (DL) lymphomas, and 6 (11%) high-grade lymphomas, 3 of which immunoblastic (IB) lymphomas, the others were small non-cleaved (SNC) lymphomas. The classification of lymphomas is summarized in Table 1.

**Table 1:** Cell types (according to NCIWF) and immunophenotypes of the 53 feline lymphomas

Lymphoma grade	Cell type	Number of lymphomas of stated immunotype		
		T-cell	B-cell	Non-B non-T
Low (*n* = 2)	DSL	1	1	0
Intermediate (*n* = 45)				
	DSC	8	6	2
	DM	4	7	0
	DL	8	7	3
High (*n* = 6)	IB	2	1	0
	SNC	0	3	0

*DSL* diffuse small lymphocytic, *DSC* diffuse small-cleaved, *DM* diffuse mixed, *DL* diffuse large, *IB* immunoblastic, *SNC* small non-cleaved

### Detection of MMTVels by nested fluorescence–PCR

**Fig. 3:**
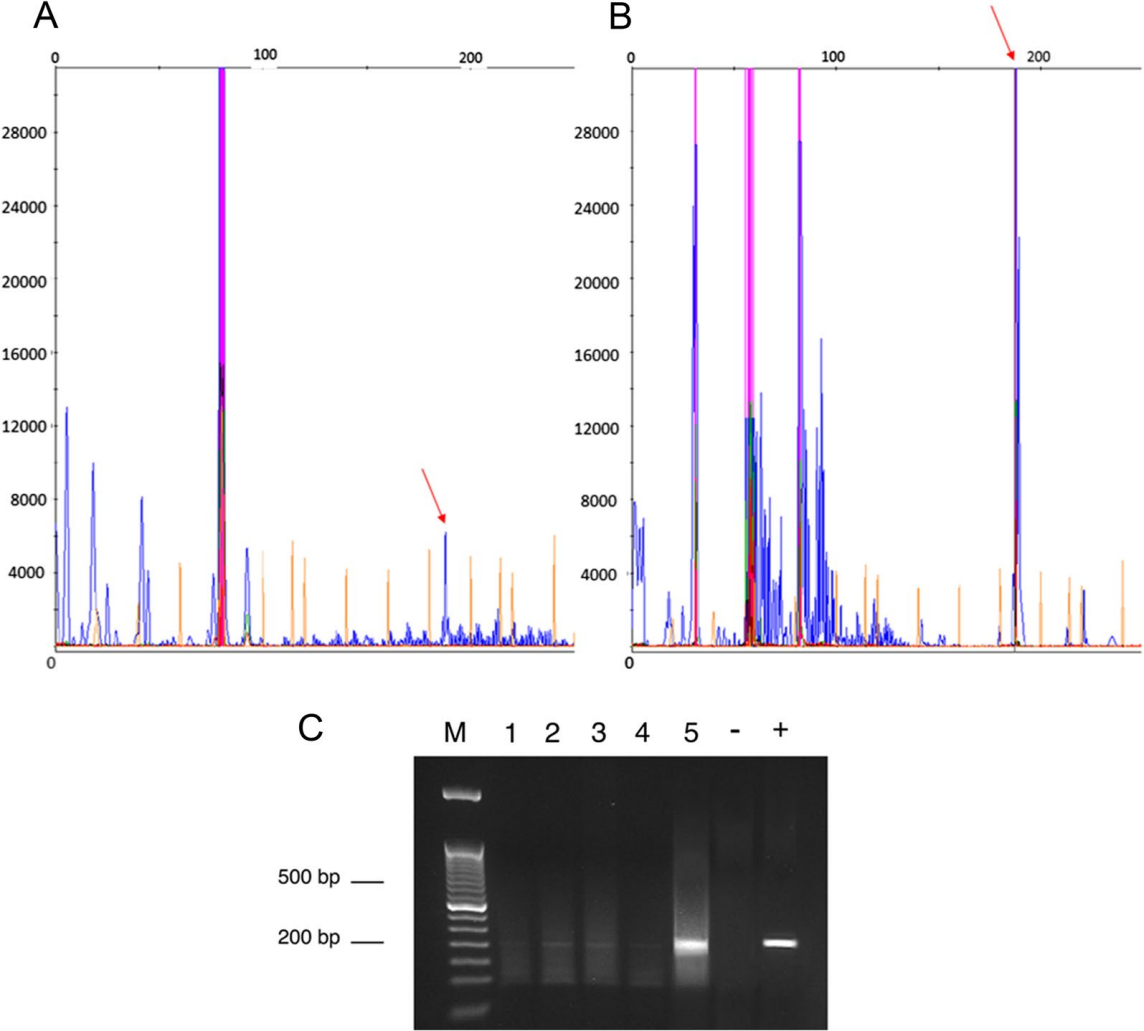
Fluorescent PCR to detect a MMTV-like sequence in feline lymphomas. **A** Fragment analyses show the blue peaks at 191 bp that represent the size of the amplified *env* fragment in sample #90722, DM B-type nasal lymphoma. **B** Fragment analyses show the blue peaks at 191 bp (red arrows) that represent the size of the amplified *env* fragment in sample #93602, SNC B-type gastrointestinal lymphoma. **C** Agarose gel electrophoresis (2%) of PCR products with a target size of 201 bp. (M): molecular weight marker; (1): ID #90722, DM B-type nasal lymphoma; (2): ID#92134, DM T-type gastrointestinal lymphoma; (3): ID#92644, DSL B-type nasal lymphoma; (4): ID#91221, DL B-type gastrointestinal lymphoma; (5): ID#93602, SNC B-type gastrointestinal lymphoma; (−) negative control; (+) positive control

Presence of *env* sequences was observed in 5 of the 53 lymphoma samples analysed (9.4%, Fig. 3).

Particularly, MMTVels positive neoplasms were 3 gastrointestinal lymphomas, 1 B-type DL lymphoma, 1 B-type SNC lymphoma and 1 T-type DM lymphoma; and 2 nasal B-type lymphomas, 1 DSC lymphoma and 1 DM lymphoma (Table 2).

**Table 2:** Classification and immunophenotype of MMTVels-positive feline lymphomas

ID	Anatomic classification	Histological classificaton	Immunophenotype
90722	Nasal	DM	B
92134	Gastrointestinal	DM	T
92644	Nasal	DSL	B
91221	Gastrointestinal	DL	B
93602	Gastrointestinal	SNC	B

*DM* diffuse mixed, *DSL* diffuse small lymphocytic, *DL* diffuse large, *SNC* small non-cleaved

As regarding the grading of malignancy, the 5 MMTVels positive samples were classified as 3 intermediate-grade lymphomas, 1 high-grade lymphoma and 1 low-grade lymphoma. None of the samples from normal feline lymphoid tissue were positive for the detection of MMTVels.

### Protein 14 immunolocalization

**Fig. 4:**
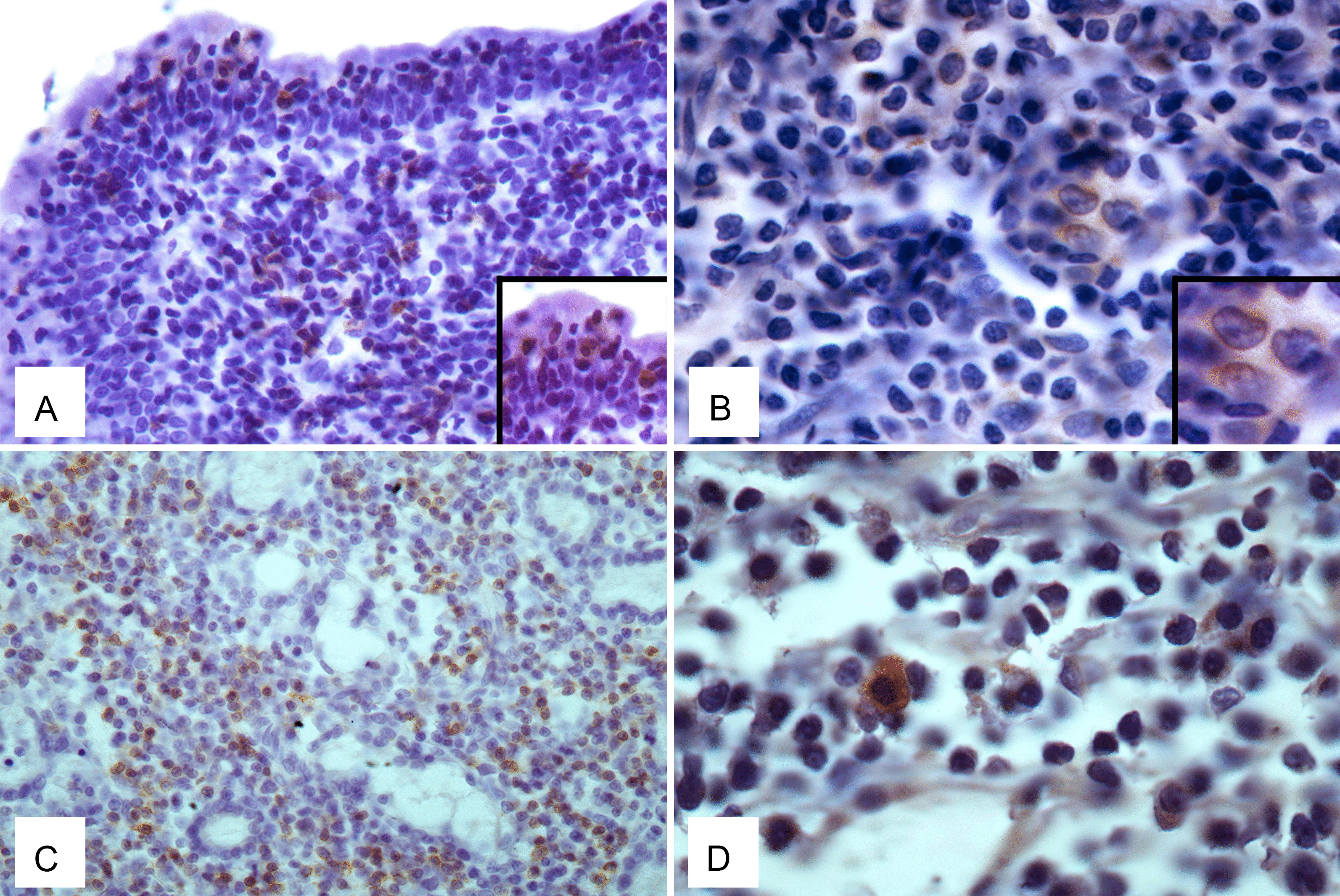
Cat MMTVels-positive lymphoma and immuno-histochemical expression of p14. **A** Gastrointestinal T-cell lymphoma, ID#92134. Infiltration of CD3-positive lymphocytes and lymphoblasts in the lamina propria of the intestine epithelium (IHC, Ob. 20×). Insert: CD3+  cells show epitheliotropism. **B** Scattered cytoplasmic expression of p14 in neoplastic lymphoblasts (IHC, Ob. 40×, ID#92134). Insert: Tumoral cells show marked anisokaryiosis and anisocytosis, indented nuclei with marginated chromatin, and prominent nucleoli (IHC). **C** Nasal B-cell lymphoma, ID #90722. Severe infiltration of CD79a-positive lymphoblasts in the submucosal layer of nasal cavities (IHC, Ob. 20×). **D** Diffuse cytoplasmic expression of p14 in tumoral lymphoblasts. Neoplastic cells show severe anisokaryosis, anisocytosis, and reticulate chromatin (IHC Ob. 40×)

P14 expression was detected exclusively in PCR-positive samples, but not in PCR-negative samples. Neoplastic lymphoid cells labelled with anti-p14 antibody were scattered and characterized by severe atypia and/or anisocytosis and anisocaryosis (Fig. 4). Positive immunostaining was cytoplasmatic.

### Supplementary anamnesis of positive subjects

MMTVels positive subjects were all FIV/FeLV negative adult, European shorthair cats. Four of the MMTVels and p14 staining positive cases were neutered males (ID #90722, DM B-type nasal lymphoma, #92134, DM T-type gastrointestinal lymphoma, #92644, DSL B-type nasal lymphoma, #91221, DL B-type gastrointestinal lymphoma), the remaining was a spayed female (ID#93602, SNC B-Type gastrointestinal lymphoma). Two cats had been adopted early in life from a cattery (ID #90722, DM B-type nasal lymphoma, and #92134, DM T-type gastrointestinal lymphoma), one subject was bought from a pet store (ID #91211, DL B-type gastrointestinal lymphoma), the remaining two were born indoors but had outdoor access (ID #92644, DSL B-type nasal lymphoma, and #93602, SNC B-Type gastrointestinal lymphoma). All the cats shared the environment with other animals, four of them with one or more cats (ID #90722, DM B-type nasal lymphoma, #92134, DM T-type gastrointestinal lymphoma, #92644, DSL B-type nasal lymphoma, and #93602 SNC B-Type gastrointestinal lymphoma), the remaining one with a dog (ID #91221, DL B-type gastrointestinal lymphoma). Of note, in two cases (ID #90722, DM B-type nasal lymphoma, and ID #93602 SNC B-Type gastrointestinal lymphoma), the cats that had shared the environment with MMTV-like positive subjects, died, in turn, because of lymphoma.

### Statistical analysis

Statistical analysis showed significant correlation between the detection of MMTVels and the anatomic localization of tumours in the nasal cavity (*p* = 0.02). There was no correlation between the presence of MMTV-like sequences and other anatomic localization, breed, sex, age, the grade of lymphoma assessed as by NCIWF classification, and the immunophenotype.

## Discussion

In this study 53 FFPE feline lypmphomas from different anatomical districts were tested for the presence of MMTV-related molecules. We succeeded in demonstrating the presence of MMTVels and p14 antigen in five cases, with a prevalence of 7%. These results are similar to those of MMTVels-positive feline mammary carcinomas found by Civita et al., 2018 (9.4%) [23] and Parisi et al. (12.5%) [24] in the same geographical area (Italy). The nearly overlapping prevalence could be further proof that the virus is involved in the carcinogenesis process of both neoplasms in feline species.

It is widely recognized that FFPE tissues are poorly indicated for molecular studies [30], and the longer the samples remain in formalin, the worse are the results [31]. In this work, it was not possible to control for this variable because the samples were collected by private clinicians and then sent to the University of Pisa for diagnosis. For this reason, our molecular investigation was carefully conducted using a combination of fluorescent PCR and subsequent fragment analysis, a procedure that has proven sensitive and robust enough to reliably detect MMTVels, minimizing issues caused by FFPE treatment [32]. The presence of viral *env* sequences was then confirmed by the detection of MMTV-like p14 expression using a previously validated immunohistochemistry protocol [19, 23, 24]. From a merely qualitative point of view, it has been possible to point out that the expression pattern of p14 in positive lymphomas did not correlate with the height of the peak for capillary electrophoresis or with the thickness of the band for gel electrophoresis, differently from what observed in a previous study on MMTV-like-positive feline mammary carcinomas [24]. P14-positive cells were scattered in all samples, if compared with p14-positive cells from MMTVels-positive feline mammary carcinomas [23, 24]. Therefore, there appears to be no correlation between the expression of viral protein and amount of viral genome copies. However, even if the group of positive neoplasms is too small to draw any definitive conclusion, the lack of correlation between protein expression and amount of viral genome could be an indicator of the reduced ability of some MMTVels-positive lymphomas to produce infectious virions, as previously inferred for mice [14, 15, 33]. Indeed, in murine MMTV-positive T-cell lymphomas, MMTV mRNAs of the *gag* and *env* genes are translated to their respective precursor proteins, but are not usually processed into mature viral proteins, so that mature viral particles are not produced [34]. However, if the presence of p14 in cells from mouse lymphoma, mammary carcinoma, and human breast cancer does imply that this protein plays a pivotal role in MMTV-associated lymphomagenesis and MMTV-associated mammary carcinogenesis [35], the same conclusions might be drawn for MMTVels-positive feline lymphoma. It may be also hypothesised that the tumour stage might influence the amount of viral genome present, as already suggested for MMTV-like sequence-positive human breast neoplasms [36].

Since p14-based immunohistochemistry is such a reliable technique that Lawson et al. [18] proposed it as a potential diagnostic tool in human breast cancer associated with MMTV, the same approach can be used to improve the consistency of data as a method independent from PCR [37, 38].

There was no correlation between the grade of malignancy and the presence of MMTVels. Studies on MMTV-like sequence-positive human breast cancer suggested that the viral infection was associated with more aggressive kinds of tumours: Ford et al. reported prevalence of MMTV-like sequences in tissues of more invasive breast cancer types from Australian women [39]; Levine et al. suggested a possible association between the presence of the MMTVels and breast tumour aggressiveness [40], and a high prevalence of MMTVels was found in Tunisian inflammatory BC patients [40]. Finally, in 2003, Wang et al. [41] found a large proportion of gestational breast cancer, known to be very aggressive and of poor prognosis [42, 43], associated with MMTV-like sequences. In 2021, Wang et al. succeeded in demonstrating a significant association between the prevalence of HMTV sequences and c-erbB-2 expression [44]. However, studies on MMTVels-positive canine and feline mammary tumours [22–24] and this study on feline lymphomas did not succeed in confirming this evidence, possibly due to the small number of samples analyzed.

The immunophenotypes of the positive lymphomas in this study were B-cell-type in four cases and T-cell-type in one case. These data are in contrast with the literature regarding MMTV lymphomas in mice, since most of the studies report that MMTV induces only T-cell lymphoma in this animal species [17, 45–50]. However, Etkind et al. found human patients with MMTV-sequence-positive B-cell lymphomas or mixed small cleaved lymphoma in their studies [51, 52]. Further studies are needed to clarify this evidence, since too little is known about the pathogenesis and the role of MMTV-like viruses in tumorigenesis in species other than mice.

Regarding the anatomic localization of the tumours, the gastrointestinal site was the most frequent for feline lymphomas in this study, in line with the literature [28]. It is, therefore, not surprising that three MMTVels-positive FL (60%) were sampled in the gastrointestinal region. On the contrary, it is noteworthy that the other two MMTVels-positive lymphomas (40%) came from biopsies from the nasal cavity, with a statistically significant correlation between the presence of a MMTVels and the nasal anatomic localization of the malignancy. The presence of MMTVels seemed, therefore, to be associated with anatomical sites directly in communication with the environment. Particularly, the amplification of viral sequences in feline gastrointestinal lymphomas could explain the role of the gastroenteric system as an entry site of infection for the virus, as demonstrated for mice in different reports. Conventional MMTV life cycle in mice begins with the ingestion of infected milk by the pups of a viremic mother, followed by infection of the intestinal epithelium. As regards feline species, it was suggested that a way to take up infectious virions might be through mouse feed, whereas the upper respiratory tract has not been hypothesised among the traditional sites of infection. However, a report from Velin et al. highlighted that the nasal lymphoid tissue of adult mice could act as an entry site for MMTV [53], and that, after adult nasal infection, the virus completed its life cycle, so that infected mice could transmit the disease. From the present data, amplification of MMTVels in tumours from nasal cavities of two cats suggests that the same hypothesis could be put forward for feline species, even if this warrants further studies.

Another interesting observation is that the MMTV-like-positive subjects in this study included four males and one female. Even if there was no statistically significant correlation between gender and the presence of MMTVels, this trend could be interesting and should be further investigated in order to understand whether male subjects are more likely to be exposed to the virus due to gender-linked behaviour.

In the attempt to use all information that could be relevant to understand the epidemiology of the virus, a deep anamnesis was collected from referring clinicians and owners of the MMTVels-positive cats. Interviews revealed that all the subjects were FIV-FeLV-negative and had a history of contact with the external environment and with other cats, if not during their entire life, at least during the first few months of life, with two of them adopted from a cattery. It is noteworthy that, among the deceased cases, two subjects spent their life together with other cats that also died of lymphoma.

From an epidemiological point of view, this association is quite interesting because it raises the suspicion of a hypothetical horizontal transmission, as already highlighted in humans [29]. However, before advancing this hypothesis, it would be useful to verify, as already observed in humans, whether the virus actively replicates and produces infecting viral particles in the feline species as well.

## Conclusions

Our study demonstrates the amplification of MMTVels and the detection of viral antigen in feline gastrointestinal and nasal lymphomas for the first time. Further studies are needed to ascertain if the association between the presence of the viral sequences and nasal localization of the lymphoid tumours may have a role in the epidemiology of a MMTV-like virus in the feline species.

## Data Availability

Histopathological material (paraffin blocks and slides) is available on request.
